# Bioinformatic analysis of related immune cell infiltration and key genes in the progression of osteonecrosis of the femoral head

**DOI:** 10.3389/fimmu.2023.1340446

**Published:** 2024-01-11

**Authors:** Xudong Duan, Fangze Xing, Jiewen Zhang, Heng Li, Yang Chen, Yutian Lei, Yiwei Zhao, Ruomu Cao, Huanshuai Guan, Ning Kong, Yiyang Li, Zidong Wu, Kunzheng Wang, Run Tian, Pei Yang

**Affiliations:** Department of Bone and Joint Surgery, The Second Affiliated Hospital of Xi’an Jiaotong University, Xi'an, China

**Keywords:** osteonecrosis of the femoral head, diagnostic biomarkers, machine learning, bioinformatics analysis, immune cell infiltration

## Abstract

**Objective:**

Osteonecrosis of the femoral head (ONFH) is a common orthopedic condition that will prompt joint dysfunction, significantly impacting patients’ quality of life. However, the specific pathogenic mechanisms underlying this disease remain elusive. The objective of this study is to examine the differentially expressed messenger RNAs (DE mRNAs) and key genes linked to ONFH, concurrently investigating the immune cell infiltration features in ONFH patients through the application of the CIBERSORT algorithm.

**Methods:**

Microarray was applied to scrutinize mRNA expression profiles in both ONFH patients and healthy controls, with data integration sourced from the GEO database. DE mRNAs were screened using the Limma method. The biological functions of DE mRNAs were explored through the Kyoto Encyclopedia of Genes and Genomes (KEGG) pathway enrichment analysis, Gene Ontology (GO) functional analysis, and Gene Set Enrichment Analysis (GSEA). Additionally, support vector machine–recursive feature elimination (SVM-RFE) and the least absolute shrinkage and selection operator (LASSO) were employed to discern diagnostic biomarkers associated with the disease. Receiver operating characteristic (ROC) analysis was utilized to assess the statistical performance of the feature genes. The validation of key genes was performed using qRT-PCR in bone tissues obtained from ONFH patients and healthy controls. Osteogenic differentiation of BMSC was then performed and detected by alkaline phosphatase staining (ALP) and qRT-PCR to verify the correlation between key genes and osteogenic differentiation. Finally, immune cell infiltration analysis was executed to evaluate immune cell dysregulation in ONFH, concurrently exploring the correlation between the infiltration of immune cells and key genes.

**Results:**

After consolidating the datasets, the Limma method revealed 107 DEGs, comprising 76 downregulated and 31 upregulated genes. Enrichment analysis revealed close associations of these DE mRNAs with functions such as cell migration, osteoblast differentiation, cartilage development and extracellular region. Machine learning algorithms further identified APOD, FBXO43 and LRP12 as key genes. ROC curves demonstrated the high diagnostic efficacy of these genes. The results of qRT-PCR showed that the expression levels of key genes were consistent with those of microarray analysis. In addition, the results of *in vitro* experiments showed that APOD was closely related to osteogenic differentiation of BMSC. Immune infiltration analysis suggested a close correlation between ONFH and imbalances in levels of Neutrophils, Monocytes, Macrophages M2, Dendritic cells activated and Dendritic cells resting.

**Conclusion:**

APOD is closely related to osteogenic differentiation of BMSCs and can be used as a diagnostic marker of ONFH. Immune cell infiltration significantly differs between controls and ONFH patients.

## Introduction

ONFH is a common clinical condition marked by the progressive deterioration, resulting in severe hip joint pain and notable functional limitations (1, 2). The disease is mainly due to the disturbance of blood supply of the femoral head caused by various reasons, which leads to the interruption of bone circulation, the death of bone active components and a series of complex pathological processes of subsequent repair. It can eventually lead to the collapse of the femoral head and degenerative changes of the hip joint. This disorder can be broadly categorized into non-traumatic and traumatic forms, depending on the underlying etiology. Traumatic ONFH is often linked to femoral neck fractures, hip dislocations, and acetabular fractures, among other causes (3–5). Non-traumatic ONFH, on the other hand, is associated with diverse etiologies, including glucocorticoid use, alcohol abuse, autoimmune diseases, and idiopathic factors (6–10). ONFH represents a progressive and destructive disease, with approximately 70% of untreated patients ultimately necessitating total hip arthroplasty (11, 12). Despite its clinical importance, the exact mechanisms governing the onset and progression of ONFH remain inadequately comprehended. Consequently, there is a pressing requirement to deepen our comprehension of the ONFH pathogenesis and to pinpoint early diagnostic biomarkers.

To date, several studies have endeavored to elucidate the underlying mechanisms involving subchondral bone in the development of ONFH. Zhang et al. postulated that intervening in the reversible stage of ONFH may confer protective effects on the femoral head by targeting subchondral bone dynamics (13). Zhao et al. noted the occurrence of partial femoral head collapse and subchondral bone fractures as integral events in the developmental process of ONFH (1), while Petek et al. highlighted compromised blood supply to the subchondral bone as a pivotal factor in ONFH pathogenesis (14). Furthermore, Jiao et al. substantiated impaired osteogenic ability in subchondral bone of ONFH through high-throughput sequencing of subchondral bone tissue (15). Despite these insights, the exploration of subchondral bone in ONFH remains relatively nascent, and the precise pathogenic mechanisms are yet to be fully elucidated. Consequently, the study of subchondral bone holds unique research value, serving as a potential avenue to discern local pathological changes in the disease.

Concurrently, an increasing body of literature has been dedicated to unraveling the complex interplay among the pathogenesis and immune cell infiltration of ONFH (16). Notably, Liang et al.’s investigation has delineated the immunological landscape by identifying IRF8 and its associated molecular components as distinctive markers of immune infiltration in steroid-induced ONFH (17). Furthermore, the research by Yu et al. underscores the substantial impact of immune cell infiltration on both the initiation and progression of ONFH, highlighting intimate correlations with specific cellular subsets (10). However, as of now, there is a lack of research specifically addressing immune cell infiltration in the cartilage and subchondral bone tissues of ONFH. Hence, investigating the immune cells infiltration in both the cartilage and subchondral bone of ONFH is very important for understanding the underlying pathogenic mechanisms of this condition.

In our study, an extensive analysis was performed by integrating microarray datasets obtained from articular cartilage tissues of ONFH patients, acquired from the Gene Expression Omnibus (GEO) database (GSE74089). Additionally, our study augmented this dataset with our own collected microarray data from subchondral bone tissues of ONFH patients. This integrative approach aimed to scrutinize the mRNA expression profiles in both cartilage and subchondral bone tissues of ONFH patients, juxtaposed with those of healthy controls. After data amalgamation, the analysis of differential mRNA expression patterns employed the CIBERSORT algorithm to identify immune cell infiltration across 22 immune cell subtypes in ONFH patients compared to their healthy counterparts. Bioinformatics analyses were then employed to delineate the functional roles of DE mRNAs, along with identifying key molecular pathways and functional networks within ONFH tissues. Ultimately, pivotal feature genes serving as molecular signatures for the diagnosis of ONFH, as well as molecular features associated with immune infiltration, by applying machine learning.

## Materials and methods

### Sample collection

The subchondral bone specimens of the femoral head of 8 patients who underwent hip arthroplasty in the Second Affiliated Hospital of Xi’an Jiaotong University were studied by using microarray. None of the patients in the study had significant metabolic bone disease, rheumatoid arthritis, systemic active infection and Paget’s disease. The samples were categorized into two cohorts based on the patient’s underlying conditions: the ONFH group (n=3, aged 57-78 years) and the control group (n=5, aged 69-85 years), comprising individuals with freshly incurred femoral neck fractures (within 24 hours of injury). **Supplementary Table 1** provides comprehensive clinical data for all patients. During the total hip arthroplasty procedure, specimens were uniformly extracted from the anterior lateral aspect of the femoral head. Ensuring consistency, the sampling area, opened in the coronal plane, was identical for both the ONFH and control groups. Subsequently, after successfully collecting the specimens, the specimens were quickly stored in liquid nitrogen at low temperature. The Ethics Committee of our hospital approved this study (Permit Number: 2021-456). All participants in this study signed informed consent forms.

### Datasets acquisition

This investigation accessed two datasets, GSE74089 and GSE7116, from the GEO database (https://www.ncbi.nlm.nih.gov/geo/). Specifically, GSE74089 employed the GPL13497 Agilent-026652 Whole Human Genome Microarray 4x44K v2 platform, encompassing cartilage samples from four healthy controls and four patients with ONFH. The training cohort was constructed by integrating our collected microarray data from subchondral bone tissues of ONFH patients with the GSE74089 dataset. In parallel, GSE7116, utilizing the GPL570 platform, comprised specimens from 11 patients with bone necrosis and 5 healthy controls, serving as the validation cohort. Probe sets without corresponding gene symbols and repeated gene symbols were removed, and the mean expression value was retained when multiple probe sets were mapped to the same gene. Subsequently, the R package “sva” was applied to systematically address and mitigate batch effects inherent across diverse datasets.

### RNA extraction, microarray hybridization and data analysis

The specific experimental methods can be found in the **Supplementary Materials and Methods**. DE mRNAs, with statistical significance between the two groups, were identified using the R package ‘Limma’. The criteria set for defining DE mRNAs were a threshold of *P* < 0.05 and log_2_|Fold change| ≥ 2.0.

### Enrichment analysis

Functional annotation and pathway enrichment analyses of DE mRNAs (DE mRNAs) were performed by using the “clusterProfiler” R package. This analysis aimed to characterize the potential biological functions through GO and KEGG pathway annotations (18). GSEA is a computational method employed to explore the enrichment status of gene sets with higher prioritization across distinct groups in terms of functions or pathways. Enrichment analysis were conducted by the “clusterProfiler” package with the threshold set at *P* value < 0.05 for the inference of functional associations.

### Screening key diagnostic markers

LASSO and SVM-RFE algorithms were employed to optimize gene selection and alleviate redundancy. Subsequently, After the differential genes are screened by the above two algorithms, the overlapping genes are considered to be discerning feature molecules. The LASSO regression analysis was conducted using the “glmnet” package (version: 4.1.2) and SVM algorithm analysis was performed utilizing the “e1071” package (version: 4.1.2) in the R software. Finally, the efficacy of these identified key feature genes was rigorously assessed through the utilization of ROC curves.

### Culture of human bone marrow mesenchymal stem cell (hBMSC) and osteoblast differentiation

</u>Human bone marrow mesenchymal stem cells were purchased from Procell (CP-H166, China) and cultured in Dulbecco’s modified Eagle’s medium(DEME; Gibco, USA) supplemented with 1% penicillin–streptomycin (Gibco, New York, USA), 10% fetal bovine serum (FBS; Gibco, USA) in an incubator containing 5% CO_2_ at 37°C. When hBMSCs reached about 60 to 70% confluence in 6-well or 12-well plates, osteogenic medium containing DMEM, 10% FBS, 1% penicillin–streptomycin, 0.1 μmol/L dexamethasone, 10 mmol/L β-glycerophosphate, and 50 μg/mL ascorbic acid substances were added to induce osteogenesis. The medium is replaced every three days until the next experiment was carried out.

### ALP staining

ALP staining was applied to hBMSCs at 3, 7 and 14 days after osteogenic induction differentiation to verify osteogenic differentiation phenotypes. BCIP/NBT ALP staining kit (C3206, Beyotime, China) was used for staining experiments. In brief, cells grown in 12-well plates were washed using PBS. After that, the cells were fixed with 4% paraformaldehyde for 30 minutes, and then washed with PBS three times. Then incubate for 2 hours at room temperature with a staining solution under dark conditions. Finally, the cells were washed with ddH_2_O to terminate the staining. Each group was compared at least three times.

### Quantitative real-time PCR (qRT-PCR)

The expression levels of mRNAs identified by microarray data were validated in a cohort comprising four ONFH patients and four controls using qRT-PCR. Total RNA extraction from hBMSCs and bone samples followed TRIzol protocols. Reverse transcription utilized the Synthesis Mix (Genestar, China), followed by qRT-PCR using the SYBR Green Fast mix (Abclonal, China). The analysis employed the 2^-ρρCt^ method. The GAPDH was considered as the internal reference gene. Genes and primers are detailed in **Supplementary Table 2**.

### Immune infiltration analysis

The CIBERSORT was applied for the computation of immune cell subtype proportions within cellular or tissue samples. Utilizing the R package “CIBERSORT,” an analysis was conducted on all DE mRNAs, with the permutation parameter set to 1000. Differences in immune cell proportions were calculated using the Wilcoxon test, and *P* < 0.05 was considered statistically significant. Subsequent correlation assessments among immune cell subtypes were performed using the R package “complot.” Moreover, Spearman analysis was employed to examine the relationships between feature genes and immune cell subtypes. Visualization utilized R packages “ggplot2” and “ggpubr”.

### Statistical analysis

The data analysis was conducted using R 4.1.2. Continuous variables underwent independent samples t-test or rank sum test. *P* < 0.05 was considered statistically significant.

## Results

### The transcriptomic profiles of mRNA were analyzed

**Figure 1:**
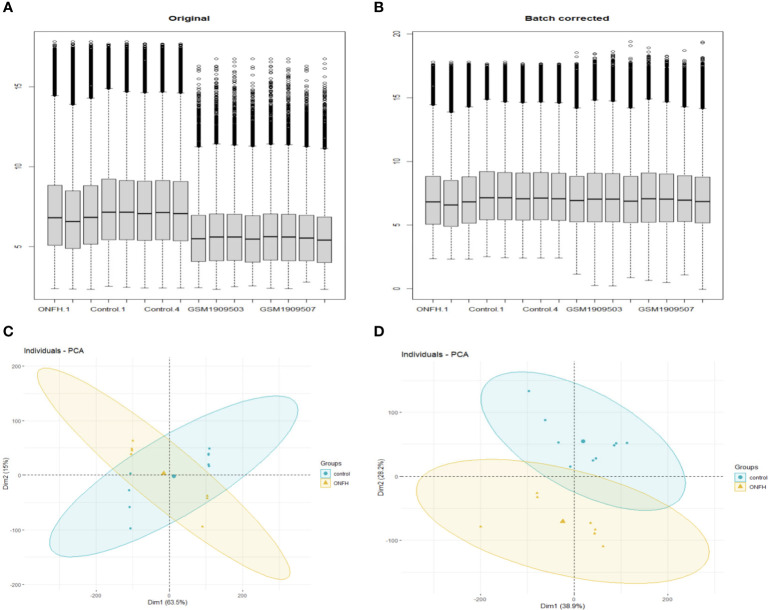
Before and after normalization of microarray data, box diagrams and PCA diagrams **(A, B)** are pre- normalization and post- normalization box charts. **(C, D)** are the results of PCA dimensionality reduction analysis before and after normalization.

**Figure 2:**
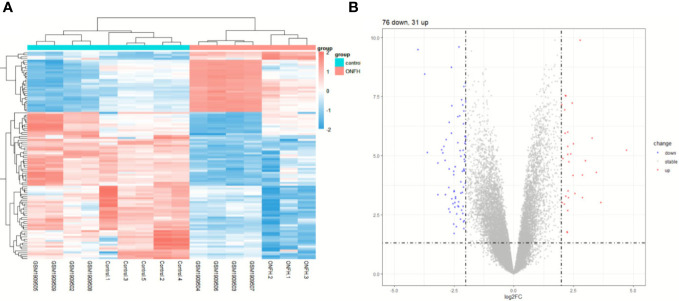
The expression profiles of DE mRNAs. Hierarchical clustering analysis was performed to display the distinguishable expression patterns of DE mRNAs **(A)**. The 107 DE mRNAs **(B)** are shown in the volcano plot.

To explore the impact of ONFH on subchondral bone, we utilized the microarray to analyze mRNA expression profiles in subchondral bone tissues obtained from ONFH patients and control patients. Both the aforementioned expression profile data and the GSE74089 expression profile data underwent standardization procedures. Batch effects were mitigated using the R package “sva” with the outcomes represented in **Figure 1**. DE mRNAs were discerned with criteria set at log_2_|Fold change| ≥ 2.0 and *P* < 0.05. In total, 107 DE mRNAs were identified, comprising 76 downregulated and 31 upregulated transcripts. Hierarchical clustering was employed to visualize distinctive expression patterns of DE mRNAs among samples, as illustrated in **Figure 2**. **Supplementary Table 3** furnishes a comprehensive catalog of the identified DE mRNAs.

### Enrichment analysis results We conducted a thorough investigation into the mechanisms underpinning ONFH by integrating expression profile data from cartilage samples

**Figure 3:**
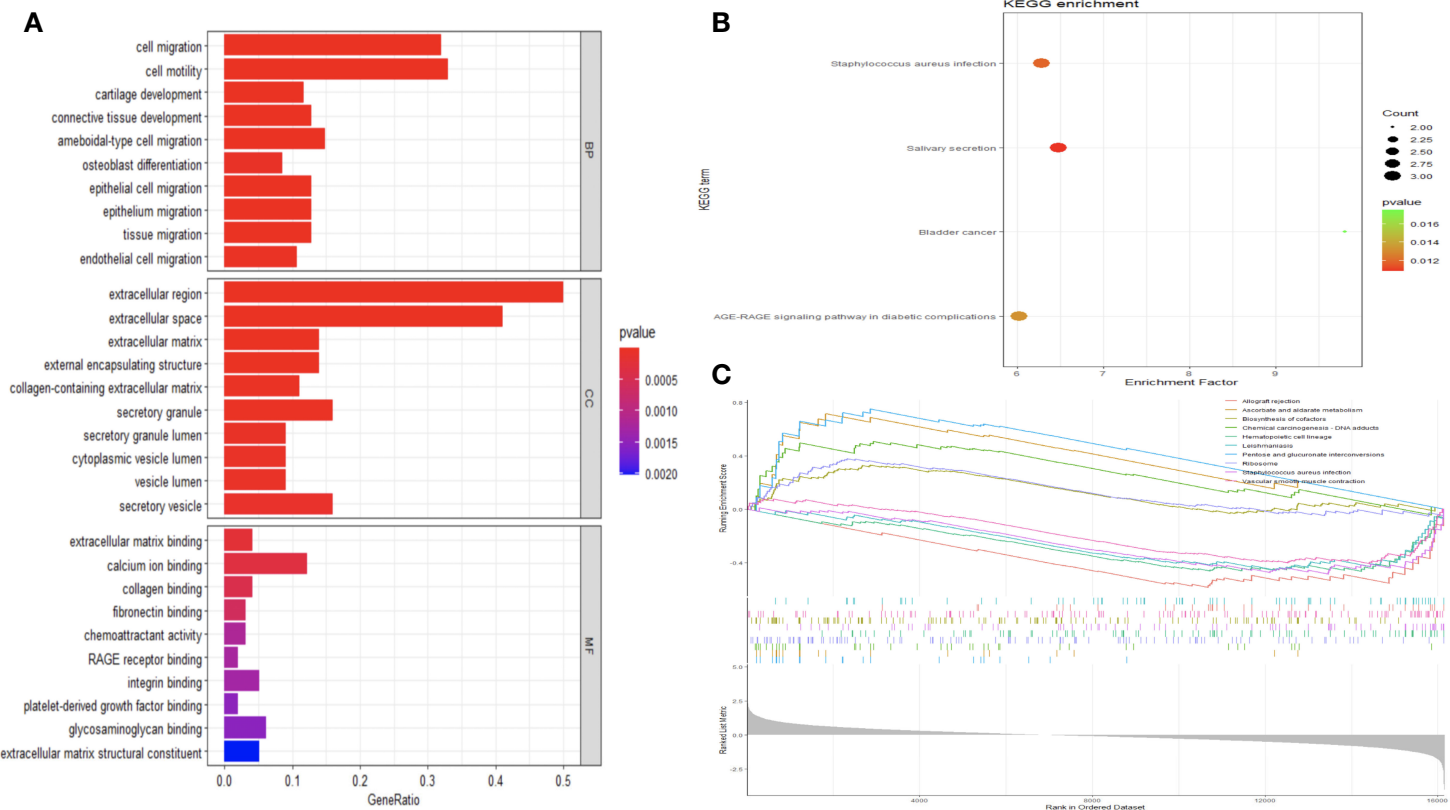
Analysis of functional enrichment in DE mRNAs. **(A)** All DE mRNAs were subjected to GO analysis. Investigation of KEGG pathways enriched with DE mRNAs **(B)**. The GSEA analysis of KEGG signaling pathways involving DE mRNAs **(C)**.

To elucidate the biological functions DE mRNAs implicated in osteochondral injury related to ONFH, we conducted an analysis comprising GO and KEGG enrichment assessments on the identified set of 107 DE mRNAs. The GO analysis unveiled the predominant functions of DE mRNAs based on GO, with enrichment observed across various functional categories such as cell migration, osteoblast differentiation, cartilage development, extracellular region, extracellular matrix, calcium ion binding, RAGE receptor binding, and other relevant entries (**Figure 3A**). The enriched pathways identified from the KEGG database predominantly encompassed salivary secretion, Staphylococcus aureus infection, AGE-RAGE signaling pathway in diabetic complications, and other pertinent pathways (**Figure 3B**). Following GSEA on the DE mRNAs, significant differences in pathways were shown in **Figure 3C**, such as pentose and glucuronate interconversions, hematopoietic cell lineage and Ribosome. The enrichment results were shown in **Supplementary Tables 4**-**6**.

### Key gene screening

**Figure 4:**
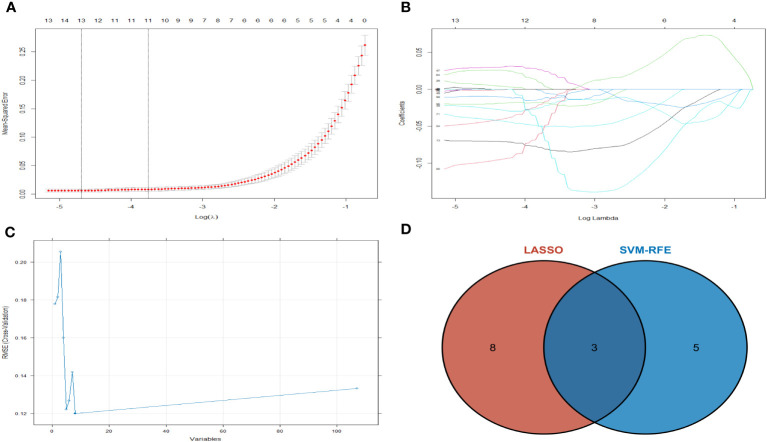
The LASSO regression and SVM-RFE model was employed. **(A)** Selection of tuning parameters (λ) by 10-fold cross-validation in the LASSO model. **(B)** The outline of the lasso coefficient profiles were plotted for 107 DE mRNAs and compared with the selected log λ values. **(C)** The gene selection by using SVM-RFE. The blue dot represents the best five variables; **(D)** DE mRNAs across 2 methods were identified.

**Figure 5:**
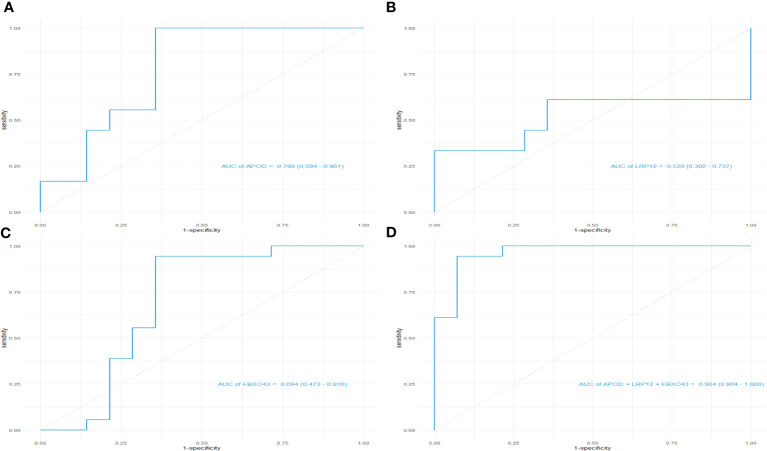
ROC curves of the diagnostic effectiveness of the key genes. **(A–D)** ROC curves of APOD, LRP12, FBXO43 and fitting three diagnostic markers to one variable; ROC = receiver operating characteristic.

Machine learning algorithms are used to further screen expressed key genes. The expression profile data of 107 differentially expressed genes were input into both the LASSO regression and SVM-RFE models. LASSO regression, implementing 10-fold cross-validation to optimize error rates, discerned 11 feature genes: APOD, CFHR3, CSN1S1, FBXO43, HBEGF, HLA-DRA, INSM1, LRP12, MRPS2, POLE and SFTA3 (**Figures 4A, B**). Simultaneously, the SVM-RFE model identified 8 feature genes: ZW10, LRP12, LYZL6, APOD, IFI6, EDN3, FBXO43 and AMY2B (**Figure 4C**). The intersection of feature genes selected by both algorithms resulted in the definitive identification of APOD, LRP12 and FBXO43 as key feature genes (**Figure 4D**). To validate the diagnostic predictive value of this gene, ROC curve analysis was employed. In the testing set (GSE7116), when APOD, LPR12 and FBXO43 were fitted into one variable, the diagnostic efficiency was reached a higher level (AUC = 0.964, 95%CI = 0.904 – 1.000) (**Figures 5A–D**). These results suggest that the APOD, LPR12 and FBXO43 possess favorable diagnostic value in distinguishing between ONFH and healthy control patients.

### qRT-PCR validation of DE mRNAs

**Figure 6:**
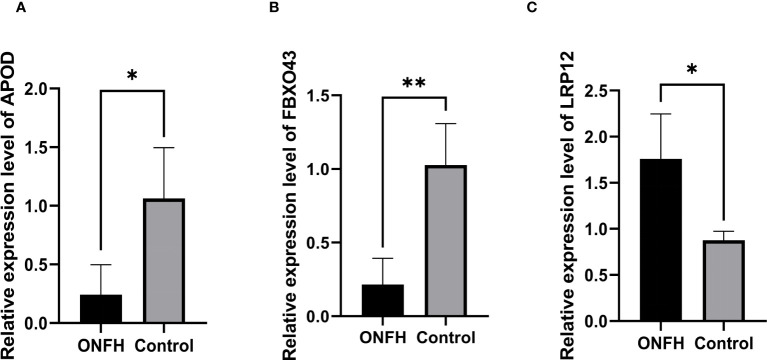
**(A–C)** The confirmation of the microarray results for mRNA through qPCR, with statistical significance at *P <*0.05. ^*^ *P*  < 0.05, ^**^ *P*  < 0.01.

To validate the reliability of the microarray data, we quantified APOD, LPR12 and FBXO43 expression in four ONFH patients and four control patients using qRT-PCR. The results of qRT-PCR showed that the expression levels of key genes were consistent with those of microarray analysis (**Figure 6**).

### Relationship between key genes and osteogenic differentiation of hBMSCs

**Figure 7:**
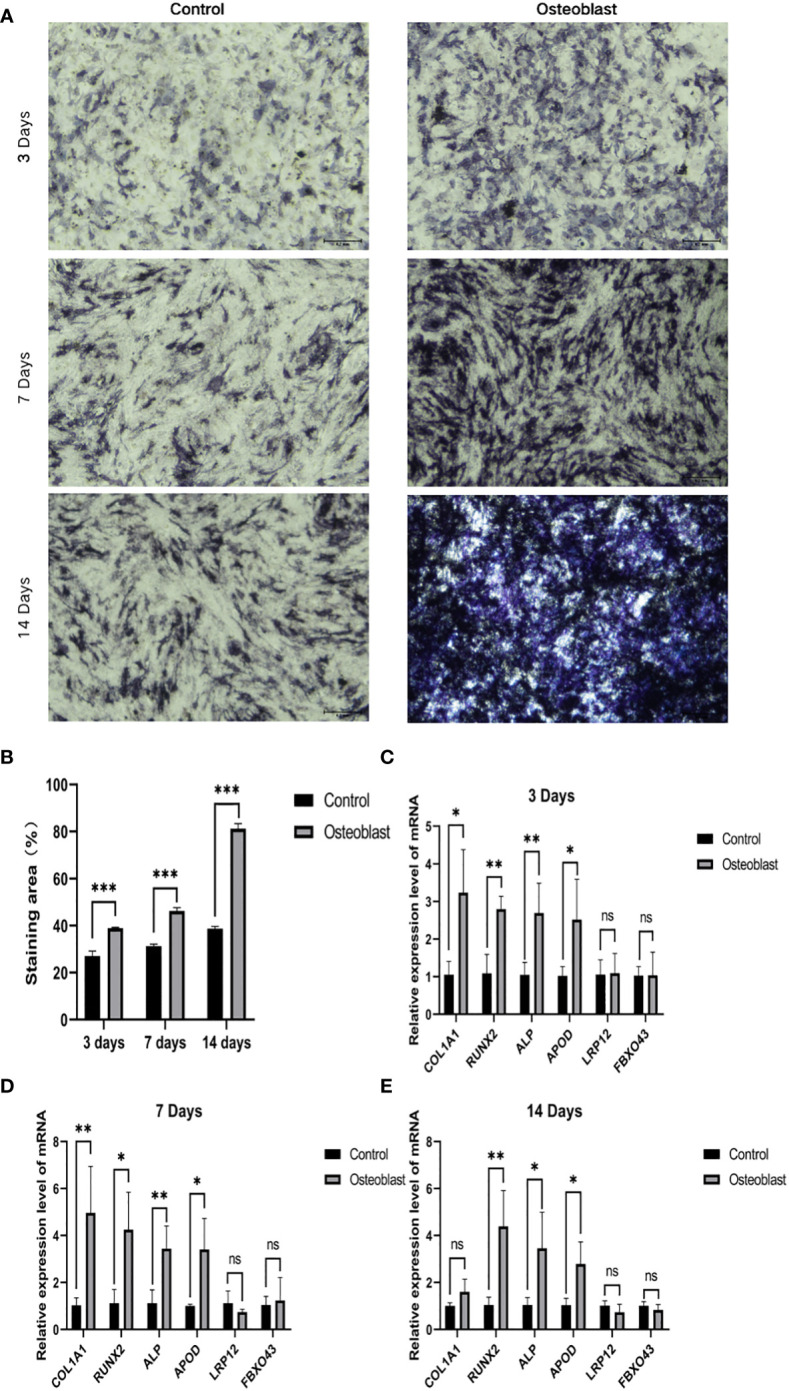
The relationship between key genes and hBMSCs osteogenic differentiation. **(A)** ALP staining on days 3, 7 and 14 of osteoblast group and control group. **(B)** Statistical analysis of ALP. **(C–E)** Expression of osteogenic genes and key genes on days 3, 7 and 14 of the osteoblast group and control group. ^*^ *P*  < 0.05, ^**^ *P*  < 0.01, ^***^ *P*  < 0.001.

In order to explore the relationship between key genes and the development of osteogenic phenotype, we used qRT-PCR and ALP staining to analyze BMSC cultured between osteogenic induction and normal medium respectively at 3, 7 and 14 days. The results of ALP staining showed that ALP activity increased gradually during osteogenic induction (**Figures 7A, B**). At the same time, the genes related to osteogenesis were detected by qRT-PCR. Compared with the control group, the expression level of related genes in osteoblast group was significantly increased (*P* < 0.05). Moreover, APOD expression levels in the osteogenic group were significantly higher than those in the control group at 3, 7 and 14 days of osteogenesis, but there was no statistically significant difference in FBXO43 and LRP12 between the two groups (**Figures 7C–E**).

### Immune cell infiltration analysis

**Figure 8:**
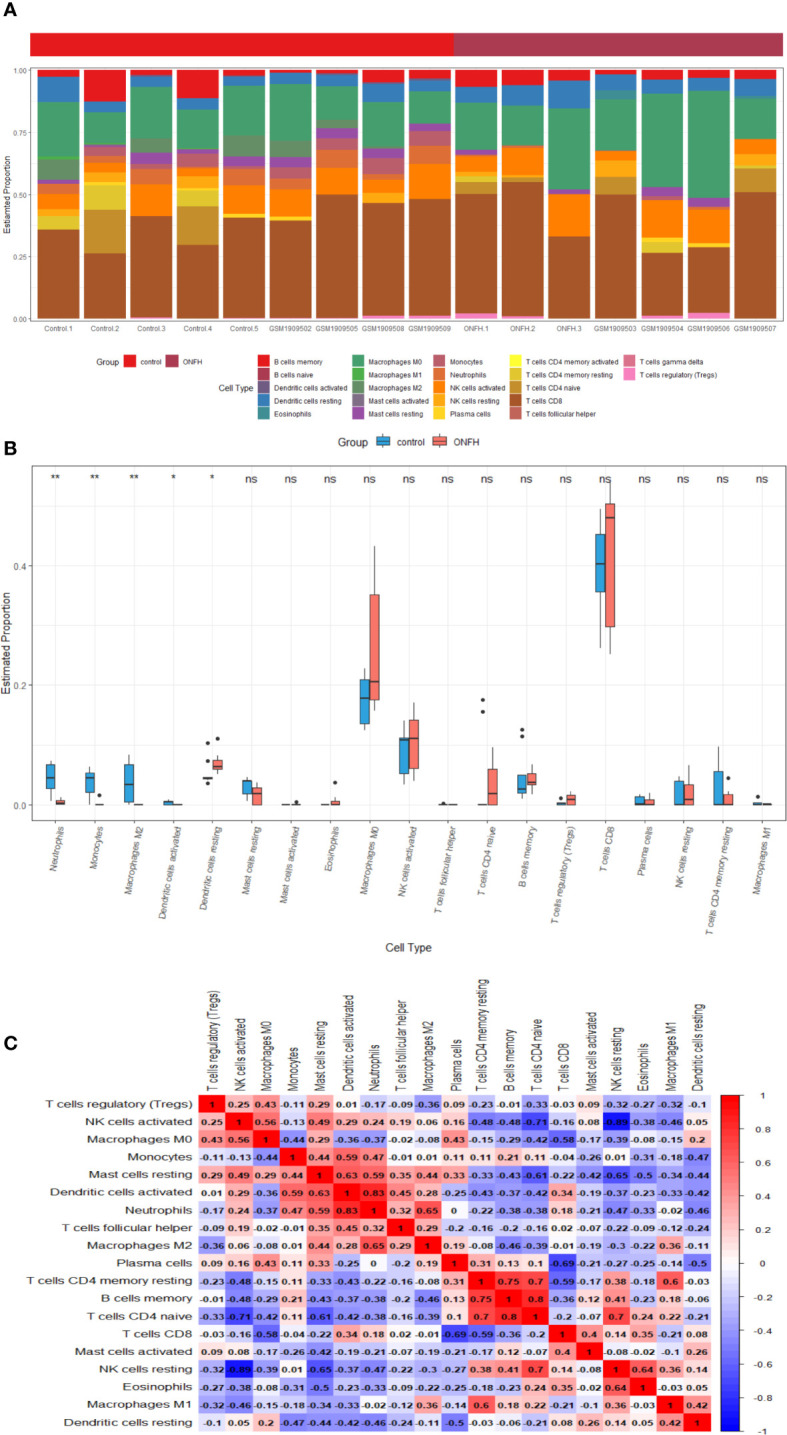
Visualization of the results of immune cell infiltration analysis **(A)** the abundance histogram of 22 subtypes of immune cells in each sample **(B)** the correlation heatmap of immune cell infiltration **(C)** the box diagram of the difference of immune cell infiltration between ONFH and healthy controls. *P < 0.05, **P < 0.01. ns, not significant.

Immune cell infiltration patterns were analyzed in the microarray data of patients with ONFH and controls, utilizing the CIBERSORT algorithm. The bar chart illustrates the percentages of 22 distinct immune cell types across all samples (**Figure 8A**). Notably, ONFH patients exhibited lower levels of Neutrophils, Monocytes, Macrophages M2 and Dendritic cells activated, coupled with elevated levels of Dendritic cells resting, in comparison to healthy controls (**Figure 8B**). Correlation analysis unveiled a negative association between Dendritic cells resting and Neutrophils (*r* = -0.46), Monocytes (*r* = -0.47), Dendritic cells resting (*r*=-0.42), Macrophages M2 (*r* = -0.11). Conversely, a positive correlation was observed between Dendritic cells activated and Neutrophils (*r* = 0.83), Monocytes (*r* = 0.59), as well as Macrophages M2 (*r* = 0.28) (**Figure 8C**).

**Figure 9:**
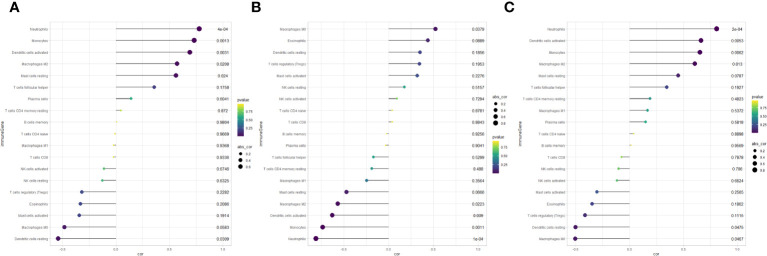
Lollipop chart of correlation analysis between key genes and immune cells. **(A)** Correlation between APOD and immune cells. **(B)** Correlation between LRP12 and immune cells. **(C)** Correlation between FBXO43 and immune cells.

In summary, discernible disparities in immune infiltration were identified. Furthermore, APOD exhibited a positive correlation with Neutrophils (*r* = 0.778, *P* < 0.001), Dendritic cells activated (*r* = 0.689, *P* = 0.003) and Monocytes (*r* = 0.732, *P* = 0.001), while demonstrating a negative correlation with Dendritic cells resting (*r* = -0.540, *P* = 0.031) (**Figure 9A**). LRP12 exhibited a positive correlation with Macrophages M0 (*r* = 0.522, *P* = 0.037), while demonstrating a negative correlation with Neutrophils (*r* = -0.808, *P* < 0.001), Monocytes (*r* = -0.736, *P* = 0.001) and Dendritic cells activated (*r* = -0.629, *P* = 0.009) and Macrophages M2 (*r* = -0.566, *P* = 0.022) (**Figure 9B**). FBXO43 exhibited a positive correlation with Neutrophils (*r* = 0.801, *P* < 0.001), Dendritic cells activated (*r* = 0.661, *P* = 0.005), Monocytes (*r* = 0.652, *P* = 0.006) and Macrophages M2 (*r* = 0.605, *P* = 0.013), while demonstrating a negative correlation with Dendritic cells resting (*r* = -0.502, *P* = 0.047) and Macrophages M0 (*r* = -0.503, *P* = 0.046) (**Figure 9C**). In conclusion, APOD, LRP12, and FBXO43 were all correlated with immune cells.

## Discussion

ONFH is an extensive and complex disease, which is characterized by microcirculation disturbance and bone marrow cell necrosis, which often leads to the destruction of trabecular structure (1). Patients with end-stage ONFH often need joint replacement. Moreover, many young patients often have to undergo revision surgery, increasing their financial burden. However, patients often delay the treatment of the disease due to the lack of clear diagnostic markers. Subchondral bone fractures commonly manifest during the mid-stage of blood supply alterations in ONFH. The critical involvement of subchondral bone in the development of ONFH has been postulated, and early intervention during the reversible phase of the disease holds the potential to mitigate or prevent femoral head collapse (13). Despite concentrated efforts, the precise molecular underpinnings of ONFH remain largely elusive. Nevertheless, a growing body of evidence emphasizes the research significance of subchondral bone in ONFH (14, 15). In our pursuit to unravel the molecular intricacies associated with subchondral bone during the development of ONFH, we utilized microarray to examine mRNA expression profiles in both ONFH patients and control groups. Concurrently, an expanding realm of research is delving into immune cell infiltration in the context of ONFH (19, 20). We conducted a thorough investigation into the mechanisms underpinning ONFH by integrating expression profile data from cartilage and subchondral bone samples, performing differential analyses, and utilizing machine learning algorithms to explore biomarkers in the disease. Additionally, the correlation between immune cell populations and ONFH was investigated through the application of the CIBERSORT algorithm, aiming to elucidate early diagnostic indicators and the fundamental mechanisms of ONFH.

An exhaustive bioinformatics analysis of gene expression profiles was initially conducted to discern key DE mRNAs associated with ONFH. A total of 107 DE mRNAs were identified, comprising 76 downregulated and 31 upregulated genes. Subsequent enrichment analysis aimed to elucidate the functional significance of all DE mRNAs, revealing their involvement in processes such as cell migration, osteoblast differentiation, cartilage development, extracellular region, extracellular matrix, calcium ion binding, RAGE receptor binding. Additionally, KEGG analysis highlighted associations of all DE mRNAs with pathways salivary secretion, Staphylococcus aureus infection and AGE-RAGE signaling pathway in diabetic complications. Further GSEA analysis indicated a concentration of prevalent genes in pathways related to pentose and glucuronate interconversions, hematopoietic cell lineage and Ribosome. Existing literature suggests that the degeneration and damage of hip joint cartilage during ONFH development increase the instability of the hip joint, thereby accelerating ONFH progression (21). Moreover, increasing evidence has demonstrated the promoting effect of the imbalance between osteoblast and osteoclast in driving ONFH progression, underscoring a close correlation between the identified DE mRNAs and ONFH (22, 23).

In recent times, machine learning algorithms, a subset of artificial intelligence, have found increased application in the realm of biomedicine. Specifically, they have been utilized for identifying diagnostic biomarkers, discovering drug targets, and elucidating biological markers (24–26). In the study, we further filtered all DE mRNAs using machine learning. Specifically, LASSO regression identified 11 key genes, while SVM-REF identified 8 key genes. The intersection of genes selected by both algorithms led to the identification of the APOD, LRP12 and FBXO43 as a diagnostic biomarker. Apolipoprotein D (APOD) is a glycoprotein. Unlike other apolipoproteins, APOD is mainly expressed in the central nervous system (27). Previous studies have reported that the expression of APOD is significantly increased during the osteogenic differentiation of human MSC (28). In addition, a study by Yu et al. demonstrated that APOD influences bone metabolism, effectively decelerating the progression of osteoporosis (29). Low density lipoprotein receptor-related protein 12 (LRP12) is a classic transmembrane protein that is widely expressed in many tissues (30). The study of Shyl et al. pointed out that LRP12 is closely related to acute myeloid leukemia (31). FBXO43 (F-box Protein 43), also called EMI2 (endogenous meiotic Inhibitor 2), many studies have shown that this molecule is closely related to a variety of cancers (32, 33).

To investigate the association between key genes and ONFH, we compared the difference in expression levels of key genes between osteogenic induction and control groups at the same time. In this study, we first detected the gene expression levels of non-inducible bone related genes such as RUNX2, COL1A1 and ALP in the osteogenic induction group, confirming that osteogenic induction is effective. At the same time, we also compared the differences in the expression of key genes between the two groups. Interestingly, the expression of APOD was significantly higher than that of the non-induced group, while the difference between the expression of FBXO43 and LRP12 was not statistically significant. Consistent with our findings, APOD is closely associated with a variety of orthopedic diseases such as osteoporosis, osteoarthritis, and ONFH (34–36). It is worth noting that APOD plays an important role in the development of osteoporosis and the osteogenic differentiation of BMSC (29). Although we did not find a link between FBXO43 and LRP12 and osteogenic differentiation in our study, this does not mean that they are not associated with the development of ONFH. They may still be able to influence the occurrence and progression of ONFH through other mechanisms such as angiogenesis. To sum up, these molecules still have high research value in ONFH, which provides a certain direction for the elucidation of ONFH mechanism in the future.

Finally, we utilized CIBERSORT to explore the immune cell infiltration in ONFH. Our observations suggest a potential association between diminished levels of Dendritic cells resting, along with heightened levels of Neutrophils, Monocytes, Macrophages M2 and Dendritic cells activated, and the initiation and progression of ONFH. These results align with prior research, such as Jiang et al.’s identification of a notable correlation between neutrophil and monocyte percentages and the incidence of ONFH (10). Additionally, existing literature underscores the substantial role played by Monocytes and macrophages, acting as osteoclast precursors, in the progression of osteonecrosis (37). Jiang et al.’s revelation of a close association between resting Mast cells and macrophages in ankylosing spondylitis combined with ONFH further substantiates our findings (38). Moreover, Wang et al.’s single-cell sequencing study, revealing a significant reduction in neutrophils and monocytes in ONFH patients, emphasizes the intricate relationship between ONFH and the dysregulation of immune cell ratios (39). Crucially, we observed a close correlation between APOD and dysregulated immune cells.

In summary, this study performed bioinformation analysis on subchondral bone tissues obtained from individuals diagnosed with ONFH and those from healthy control subjects. The expression profile data were subsequently integrated with microarray data from cartilage samples of ONFH patients. Furthermore, employing machine learning algorithms, we performed additional filtering of the expression profile data to identify key genes. We subsequently verified the correlation of key genes with the development of ONFH through *in vitro* experiments. Finally, an exploration was conducted to elucidate potential relationships between the occurrence of ONFH and immune cell infiltration. To date, the demand for the prevention and treatment of ONFH has been increasing. However, research on the association between ONFH and immune cell infiltration remains limited. The results of this study offer novel insights into the molecular mechanisms underlying ONFH and contribute valuable information for identifying potential molecular markers associated with this condition.

Nonetheless, this study is not without its limitations. Firstly, the sample size in our clinical cohort is relatively modest, potentially introducing bias into the analysis. Secondly, the molecules validated have not been confirmed by *in vivo* studies. Additional investigations are imperative to substantiate the roles attributed to the identified molecules in ONFH as delineated in this study.

## Conclusion

This study utilized microarray to characterize mRNA expression profiles in ONFH patients and analyzed the functional pathways and roles of differentially expressed genes. The machine learning algorithm identified APOD, FBXO43 and LRP12 as distinctive gene associated with ONFH, and its validation was performed through ROC curves and vitro experiments. The results show that APOD is closely related to osteogenic differentiation of BMSCs and can be used as a diagnostic marker of ONFH. Simultaneously, we observed a significant correlation between key genes and immune cells. Moreover, marked variations in the abundance of these immune cells were observed between ONFH patients and the control group. Consequently, our findings contribute to a deeper comprehension of ONFH pathogenesis and offer identifying molecular markers for ONFH diagnosis and treatment.

## Data availability statement

The datasets presented in this study can be found in online repositories. The names of the repository/repositories and accession number(s) can be found in the article/**Supplementary Material**.

## Ethics statement

The studies involving humans were approved by the ethics committee of the second affiliated Hospital of Xi’an Jiaotong University. The studies were conducted in accordance with the local legislation and institutional requirements. The participants provided their written informed consent to participate in this study.

## Author contributions

XD: Conceptualization, Formal Analysis, Writing – original draft, Writing – review & editing. FX: Data curation, Writing – original draft. JZ: Data curation, Writing – original draft. HL: Data curation, Formal Analysis, Validation, Writing – original draft. YC: Data curation, Formal Analysis, Writing – original draft. YTL: Data curation, Formal Analysis, Writing – original draft. YZ: Data curation, Formal Analysis, Writing – original draft. RC: Data curation, Formal Analysis, Writing – original draft. HG: Data curation, Formal Analysis, Writing – original draft. NK: Data curation, Formal Analysis, Writing – original draft. YYL: Data curation, Formal Analysis, Investigation, Writing – review & editing. ZW: Data curation, Formal Analysis, Writing – original draft. KW: Data curation, Formal Analysis, Writing – review & editing. RT: Writing – review & editing. PY: Data curation, Funding acquisition, Resources, Supervision, Writing – original draft.
