# Corrigendum: Bioinformatic analysis of related immune cell infiltration and key genes in the progression of osteonecrosis of the femoral head

**DOI:** 10.3389/fimmu.2024.1410267

**Published:** 2024-04-16

**Authors:** Xudong Duan, Fangze Xing, Jiewen Zhang, Heng Li, Yang Chen, Yutian Lei, Yiwei Zhao, Ruomu Cao, Huanshuai Guan, Ning Kong, Yiyang Li, Zidong Wu, Kunzheng Wang, Run Tian, Pei Yang

**Affiliations:** Department of Bone and Joint Surgery, The Second Affiliated Hospital of Xi’an Jiaotong University, Xi’an, China

**Keywords:** osteonecrosis of the femoral head, diagnostic biomarkers, machine learning, bioinformatics analysis, immune cell infiltration

In the published article, there was an error. GSE74089 included 4 ONFH patient cartilage samples and 4 normal cartilage samples and not “subchondral bone specimens”.

A correction has been made to **Materials and methods,** *Datasets acquisition*. This sentence previously stated:

“Specifically, GSE74089 employed the GPL13497 Agilent-026652 Whole Human Genome Microarray 4x44K v2 platform, encompassing subchondral bone specimens from four healthy controls and four patients with ONFH.”

The corrected sentence appears below:

“Specifically, GSE74089 employed the GPL13497 Agilent-026652 Whole Human Genome Microarray 4x44K v2 platform, encompassing cartilage samples from four healthy controls and four patients with ONFH.”

The authors apologize for this error and state that this does not change the scientific conclusions of the article in any way. The original article has been updated.

