# Supplementary material for: Bioinformatic analysis of related immune cell infiltration and key genes in the progression of osteonecrosis of the femoral head
Source: Front Immunol. 2024 Jan 11;14:1340446. doi: 10.3389/fimmu.2023.1340446 (PMC10811953; doi:10.3389/fimmu.2023.1340446)
Supplement: Supplementary file 1 [file DataSheet_1.docx]

Supplementary Material

# Supplementary Materials and methods

RNA extraction, microarray hybridization

Liquid nitrogen is used to store subchondral bone samples from fresh ONFH patients and healthy controls. RNA extraction and purification procedures were executed utilizing the Trizol reagent (Invitrogen) and the RNasey Mini Kit (Qiagen). The quantification and quality assessment of RNA was conducted by using the NanoDrop(ThermoFisher).For sample labeling and array hybridization, the Agilent One-Color Microarray-Based Gene Expression Analysis protocol (Agilent Technology) was employed with slight adaptations. Finally, the microarray is used to measure the expression profile data.
