# Supplementary material for: Bioinformatic analysis of related immune cell infiltration and key genes in the progression of osteonecrosis of the femoral head
Source: Front Immunol. 2024 Jan 11;14:1340446. doi: 10.3389/fimmu.2023.1340446 (PMC10811953; doi:10.3389/fimmu.2023.1340446)
Supplement: Supplementary file 3 [file Table_2.docx]

## Supplementary Table S2. qRT-PCR Primers

| Gene | Primer |
| --- | --- |
| GAPDH | F:5'GACAGTCAGCCGCATCTTCT3'  R:5’GCGCCCAATACGACCAAATC3’ |
| APOD | F:5' CTTTGAGAATGGACGCTGCA 3’  R:5’ TTCTCATAGTCGGTGGCCAG’3 |
| LPR12 | F 5′-GTGTGTTTGAAAGTTGGGTGTGTG-3′ |
|  | R 5′-CTATGACGGCAGCAGTGATGAC-3′ |
| FBXO43 | F 5′-CTCCGATAAGTAATCTTGTGGC-3′ |
|  | R 5′-CTTGTCTTTCTTATGGTGTCCC-3′ |
| ALP | F 5′- ACCACCACGAGAGTGAACCA-3′ |
|  | R 5′- CGTTGTCTGAGTACCAGTCCC-3′ |
| COL1A1 | F 5′- AGACAGTGATTGAATACAAAACCA-3′ |
|  | R 5′- GGAGTTTACAGGAAGCAGACA-3′ |
| RUNX2 | F 5′- CAGTGCAGGGTCCGAGGT-3′ |
|  | R 5′- AGATGATGACACTGCCACCTCTG-3′ |
